# Hydrophobia

**Published:** 1830-10-01

**Authors:** 


### Periscope;

OR,

### CIRCUMSPECTIVE REVIEW.

" Ore trahit quodcunque potest, atque addit acervo."

I.

### HYDROPHOBIA.

1. Remarks on Hydrophobia. By J. Murray, F.S.A.

2. On Canine Madness, &c. By W. Youatt, V.S. &c.

When the lake is agitated by the storm, sand and mud will be raised from the bottom, and float, for a time, on the waves. When Ætna or Vesuvius is convulsed by subterranean fires, stones will rise into the air, and ashes will wing their flight to Egypt, Constantinople, or Africa. So, during the late epidemic cynophobia, a host of speculators rushed into print, and the Times, the Herald, and the Lancet teemed with proposals, theories, and therapeutics, not one of which was worth the lamp-black and oil expended on the printer's types in their progress through the press. It is not our intention to notice these crude productions of excited imaginations. One example will be sufficient. Mr. Jenkins has proposed excision of the largest and nearest *nerve* to the hydrophobic wound! The reasoning on which this proposal is founded, amused us a good deal. As the disease is sometimes many months, and generally several weeks dormant after the receipt of the poison, Mr. J. infers that the *absorption* goes on through the medium of the nerves, and not through the medium of the absorbents or blood-vessels! He does not give us any reason why the virus of the dog should refuse to take the usual routes, and insist on marching with a snail's pace along the nerves. He does not, he cannot offer us any proof respecting the period of absorption at all. How does he know the time necessary for the poison's residence in the system at large? Does the matter of small-pox remain in the wound where it is inoculated for several days till the constitutional symptoms commence? Does the contagion of plague, or typhus, or measles remain on the air passages, or the skin, or the nares, for a fornight or three weeks, before absorption, and constitutional disturbance? Every specific poison appears to have its own period of *incubation*, if we are allowed the expression, and where its habitation is, or what its operation is during that period, we are totally ignorant, and probably will remain so. But the proposal of cutting out the most proximate nerve to the wound is, we apprehend, one of the wildest aud most absurd speculations which we have yet heard. The whole question respecting the pathology and therapeutics of hydrophobia remains in statu quo—namely, in utter darkness—and the prophylaxis is nearly in the same state. Excision is the preventive which rests on the basis of facts—suction is doubtful—and all other prophylactics are speculations, without the shadow of proof.

Nevertheless we shall give one of the latest proposals which have been made, because the author is a sensible and clever man, though not strictly of the medical profession—Mr. Murray, the chemist. The preventive which this gentleman proposes, in addition to the usual local means as respects the bite, is an atmosphere of chlorine.

" He might also for sometime wear the portable voltaic instrument of Bansford, to which at any rate there can be no reasonable objection; and it may also, on a review of the nature of the disease, he deemed interesting, to encase the body in oiled silk or otherwise, and by this means be enabled to surround the cuticular surface with an atmosphere of chlorine, disengaged in the usual way from a retort, containing its ingredients, the beak of which is inserted into the envelope. We are anxious to employ the entire apparatus of safety, and be secure at all hazards by doubling the means of precaution, though part may be supposed superfluous and redundant."

"Whether the nervous system be primarily or secondarily affected, it is extremely advisable to apply voltaic electricity to the patient, *ab initio*. The pensile galvanic pile is a convenient mode of excitement, and is promptly renewed; the organs concerned in respiration should be included in the circle formed by the conducting wires, and afterwards the brain. Our chief reliance would be on an atmosphere of chlorine, applied in the way already described, to the surface of the body, so that the system should be charged with it. The next remedial measure is to impregnate the atmosphere which the patient breathes, either with chlorine or with the vapour of red fuming nitrous acid, placed near, and the latter would less irritate the lungs; the inflammatory action might be subdued by the frequent internal exhibition of chlorate of potassa, in doses repeated at frequent intervals, of from four to eight grains: we know oxygen has been suggested by way of trial in this disease, but the important facts established by Count Morozzo, Allen and Pepys, and more recently by Mr. Broughton, give us no reason to expect relief from that quarter. Animals soon die in pure oxygen, and the gas, it is interesting to remark, suffers no chemical change.

We have already adverted to the absorption by the pores of the skin, of carbonic acid gas, and the experiments of Dr. Edwards, in the 'Annales de Chimie et de Physique,' (January, 1819,) corroborate this by analogy. It is well known that water applied to the surface of the skin allays thirst, as immersion in salt water: and in the desert the traveller has revived from that fatal state into which he was fast sinking by skins of water being thrown on the body. In Mr. Broughton's interesting experiments,* we find in the case of animals that perished in chlorine gas, the peculiar odour was perceptible throughout the structure of the lungs, a full proof that the gas had passed the epiglottis, and entered the system by the air passages: the tissue of the lungs was also dyed yellow, and we are not acquainted with any aerial agents whose power to subdue inflammation, &c. of this kind, surpasses chlorine, and the vapour of nitric, and nitrous acid gas: the remedy must be introduced through the medium of respiration, in order to bring it in immediate contact with the mucous membrane of the bronchiæ, the seat of active inflammation.

Frictions of iodine externally in the region of the lungs may be also esteemed a remedial measure worthy of trial. When the skin, *however dry*, is brought in contact with chlorine there is the sensation of a considerable increase of temperature immediately produced, though the thermometer is completely unaffected in the case: this fact was first pointed out by Dr. Hare, of Philadelphia, and we discovered a precisely similar phenomenon with nitrous acid gas. (Philosophical Mag. and Journal, vol. 60, p. 100.) This fact proves a very determinate and specific action on the skin, and it must not be forgotton that comparatively dry chlorine will act a very different part from the same gas held *in solution in water*,—*hygrometrically* as well as chemically. In the case of chlorate or oxymuriate of potassa, its medicinal efficacy is connected immediately with the circulation: it will soon give evidence that it is so by lowering the pulse without any prostration of physical strength. It was exhibited on our suggestion in a case of epilepsy, and the blood that was subsequently drawn from the patient was remarkably bright, being of a brilliant red. Its effect is frequently instantaneous, and

* See Mr. Brande's Journal, for 1830, p. 15, &c.

the morbid state of the blood in hydrophobia seems to warrant us to expect some important relief from its exhibition. M. Ségalas D'Etchepare has shown that besides a direct and sympathetic action on the organic solids it exercises a very manifest one on the blood, and consequently over the whole economy through the medium of absorption; and had Majendie's injections of tepid water into the veins, held *chlorate of potassa in solution*, the case might have been happily altogether different."

The above extract contains the gist of Mr. Murray's proposal, and to which we see no possible objection. It is a much more rational tentative than that of Mr. Jenkins; but whether it may prove a whit more successful, is yet to be decided by facts.

---

Mr. Youatt's work on canine madness is a sensible and useful performance, being the result of much practical experience. In the following extract, we apprehend that even this experienced observer has assumed more than is capable of proof in regard to the dormant period of the hydrophobic virus—or rather the locality of the poison during that period.

"An animal or a human being is bitten by a rabid dog; and a certain portion of the poison is received into the wound. It produces no immediate irritation; there is nothing to indicate its presence. Some old writers, indeed, recommend us to place the fresh leaves of rue on the wound. If they retain their colour, it is not envenomed. If they change to a violet colour, the rabid poison has been introduced. They likewise tell us to rub a bit of bread in the blood or fluid discharged from the wound. If there be no danger, a dog will readily eat of it; if it be contaminated with the virus, he will refuse it with howling.

These fooleries are now despised. There is nothing to indicate the presence of the virus. The wound heals, as would another wound, according to its situation, magnitude, laceration, and the constitution of the patient. Days, and weeks, and months pass, and not the slightest circumstance occurs to indicate the impending danger.

What then is this virus? It has never been analysed. That would be a process difficult to accomplish. It is not diffused through the air, nor communicated by the breath, nor by any effluvia, nor even by actual contact, if the skin be sound. It must be received into a wound; or it must come in contact with some tissue or nervous fibril; and there it lies dormant for a considerable but uncertain period, and longer in some animals than in others.

*It remains perfectly undecomposed. The absorbents are actively at work in removing every thing around. The capillary vessels are depositing fresh matter, but it seems to remain the same. Whatever else is useless, or would be injurious, is taken up, and the tissue or the fibril on which the virus rests, is modified or changed; but this extraneous and fatal body bids defiance to all the powers of nature.*

*It enters not into the circulation, or it would necessarily undergo some modification in its passage through the innumerable minute vessels and glandular bodies which are scattered through the frame.* It would excite some morbid action; or if it were not thus employed, or in the purposes of renovation or nutrition, it would be speedily ejected.

It lies for an uncertain period dormant; but at length, from its constant presence as a foreign body, it may have rendered the tissue or nervous fibril more irritable and susceptible of impression; or it may have attracted and assimilated to itself elements from the fluids that circulated around it, and thus increased in bulk; and at length, according to a law of chemistry, supplied by quantity that which it wanted in strength of affinity.

Whatever be the *modus operandi*, the parts in contact with the virus at length respond to the stimulus applied to them. The cicatrix generally begins to itch, and inflammation spreads around it. The diligent licking of some part where the mark of a bite can be traced, is an early and frequent symptom of rabies in the dog. The absorbents are now called into more powerful action. They begin to attack even the virus. A portion of the morbific matter is taken up and carried into the circulation, and disease and death ensue."

If the virus lurks so long in the wound, how is it that "excision of the part has frequently failed," according to Mr. Youatt's own confession? The following explanation is, to us, very unsatisfactory.

"The knife may penetrate the deep and tortuous recess of the wound, in which the virus is lodged, and then its track will be empoisoned. Or if the incision be freely made round the wound, and does not penetrate into it, the blood will follow the knife; a portion of it will enter into the wound inflicted by the dog; it will come in contact with the virus; it will be contaminated; it will overflow that cavity; it will be received into the new incision, and it will carry with it the seeds of disease and death."

Mr. Youatt, therefore, abandons excision, and has recourse to the nitrate of silver as a caustic. The stick is to be sharpened to a point, and every sinuosity of the wound is to be searched and penetrated. The eschar having sloughed off, he advises the re-application of the caustic.

"It is painful to speak of one's-self; but I may, perhaps, here be permitted to say, that I have been bitten four times by dogs decidedly rabid. At each time I freely applied the caustic to the wound; and I am living to the present day. I have operated on more than four hundred persons, all bitten by dogs, respecting the nature of whose disease there could be no question.* I have not lost a patient. One poor fellow died of fright, but not one became hydrophobous. To what can I so naturally attribute this, as to some chemical affinity between the nitrate and the virus, by which an insoluble and inert compound is formed?"

* "I am bound to add, that one of the surgeons of St. George's Hospital told me, that since his connexion with that establishment, he and his colleagues had operated on more than as many thousands, bitten by dogs (he could not say that all of them were rabid), and he was not aware that one of them had been lost. This, at least, is most consolatory, whatever may become of my theory of the caustic."

